# Development and validation of a patient no-show predictive model at a primary care setting in Southern Brazil

**DOI:** 10.1371/journal.pone.0214869

**Published:** 2019-04-04

**Authors:** Henry Lenzi, Ângela Jornada Ben, Airton Tetelbom Stein

**Affiliations:** 1 Serviço de Saúde Comunitária–Grupo Hospitalar Conceição, Porto Alegre, Brazil; 2 Department of Health Sciences, Vrije Universiteit Amsterdam, Amsterdam, The Netherlands; 3 Departamento de Saúde Coletiva, Universidade Federal de Ciências da Saúde de Porto Alegre, Porto Alegre, Brazil; Universidad Miguel Hernandez de Elche, SPAIN

## Abstract

Patient no-show is a prevalent problem in health care services leading to inefficient resources allocation and limited access to care. This study aims to develop and validate a patient no-show predictive model based on empirical data. A retrospective study was performed using scheduled appointments between 2011 and 2014 from a Brazilian public primary care setting. Fifty percent of the dataset was randomly assigned to model development, and 50% was assigned to validation. Predictive models were developed using stepwise naïve and mixed-effect logistic regression along with the Akaike Information Criteria to select the best model. The area under the ROC curve (AUC) was used to assess the best model performance. Of the 57,586 scheduled appointments in the period, 70.7% (n = 40,740) were evaluated including 5,637 patients. The prevalence of no-show was 13.0% (n = 5,282). The best model presented an AUC of 80.9% (95% CI 80.1–81.7). The most important predictors were previous attendance and same-day appointments. The best model developed from data already available in the scheduling system, had a good performance to predict patient no-show. It is expected the model to be helpful to overbooking decision in the scheduling system. Further investigation is needed to explore the effectiveness of using this model in terms of improving service performance and its impact on quality of care compared to the usual practice.

## Introduction

Patient no-show is defined as a scheduled appointment that the patient neither attended or canceled on time to be reassigned to another patient [1,2]. It implies ineffective use of human and logistic resources in a scenario where the demand for health care is greater than the supply. Beyond that, the patient non-attendance could compromise the core principles of primary care: the accessibility and the continuity of care [3]. Whenever a patient misses an appointment, two patients fail to access health care: the no-show patient and the patient who could not book an appointment. Also, patient non-attendance leads to a discontinuity of care, which is associated with worsening of health outcomes such as increasing of hospitalization rates due to exacerbations of chronic conditions [4–6]. There are also additional costs, e.g., time spent on mitigation strategies and health care staff idle time [7].

The prevalence of no-show varies worldwide. It has been shown to be higher in low income and developing countries [1]. Dantas *et al*., in a literature review, found the second highest no-show prevalence in South America (27.8%) after the African continent (43.0%) [1]. In Brazil, despite the shortage of data on this issue, studies have reported no-show rates of 48.9% at primary care [8] and 34.4% at specialized point-of-care service [9]. It has been described that decreasing no-show rates could have resulted in substantial savings especially in universal health care systems[10]. For instance, in the National Health Service of the United Kingdom, a reduction in no-show prevalence from 12% to 10.8%, would decrease the annual public expenses by 10% [10].

Given the aforementioned, factors associated with patient no-show have been investigated to provide insights about target interventions. Young age and previous patient non-attendance have been consistently reported [11–16]. An association has also been found between longer lead time (the time between the scheduling and the appointment) and higher no-show rates [1]. Other factors are related to the type and severity of the problem; sociodemographic conditions; appointment period of the year and distance to service [17]. Since these factors may vary across populations and health care services, a common set of universal determinants is unlikely to be found. Hence, this implies that it behooves each service to investigate local predictors, to tailor actions to address the issue. Based on that, no-show predictive models have been developed to optimize the scheduling process and service performance, but mainly in developed countries setting [17–21].

To the best of our knowledge, there have been no published studies about developing no-show predictive models based on data from a Brazilian public health care scenario. Therefore, the present study aims to explore the factors associated with a no-show at a Brazilian primary care setting and to develop and validate a patient no-show predictive model based on empirical data.

## Materials and methods

### Study design

A retrospective study was performed based on the scheduled appointments registered in the scheduling system of a public primary care service of the Grupo Hospitalar Conceição between November 1, 2011, and March 31, 2014. Patient record numbers were irreversibly replaced by a sequence of random characters (fully anonymized) before the analysis. The study was approved by the Ethics Committee of the Grupo Hospitalar Conceição (number 2.349.672). Ethics committee waived the requirement for informed consent.

### Patient no-show predictors

The no-show predictors were chosen based on literature, on the experience of the primary care service team and considering the availability of data in the scheduling system. The unit of analysis was the scheduled appointment. No-show was defined as non-attendance at the appointment day until the closing time of the service at 6 pm. For each unit of analysis, the following variables were available in the scheduling system: patient record number; age (years); gender (male/female); self-reported race/ethnicity registered in the scheduling system according to the Ethno-Racial Characteristics of the Brazilian Population [22] and dichotomized as white and non-white; appointment day; date and time of the scheduling; date and time of the appointment; appointment shift (morning or afternoon); appointment weekday; appointment month; appointment attendance (attendance/no-show); health professional categories (nursing, dentist, general practitioner, pharmacist, nutritionist, psychologist, social worker and oral health technician) and types of appointment–S1 Table.

For each appointment scheduled, the following metrics were calculated: “lead time” (the time between the scheduling and the appointment in days); “waiting time” (difference between the appointment time and the time it was held in minutes); “patient previous attendance” (number of times the patient has attended the previous appointments) and “patient previous same-day appointment” (number of times the patient had previous same-day appointments). A dichotomous variable “same-day appointment calculated” was generated for those appointments scheduled and held on the same day (1) or not (0) to verify consistency with the category “same-day appointment” of the variable “types of appointment”–S1 Table.

Observations were included if they did not fulfill the exclusion criteria. Observations were excluded and deleted from the analysis if: 1) the information on the outcome was not registered, and 2) there was no possibility to derive metrics from the available data.

### Statistical analysis

#### Descriptive analysis of the dataset

First, a descriptive analysis of variables was performed by the categorical outcome: attendance (0) and no-show (1). Means and standard deviations were calculated for normally distributed variables and medians and quartiles for nonparametric variables. Frequencies and percentages were calculated to describe categorical variables. The normality of continuous variables was evaluated by the D’Agostino skewness test [23]. All variables were included as predictors in the process of model selection. Analyses were performed in R software, version 3.5.2.

#### Model development and selection

Afterwards, the dataset was randomly divided into two subsets by using the Caret R package [24]: 1) 50% of the dataset was assigned to develop the logistic regression model (training subset) and 2) the remaining 50% was assigned to validate the model (validation subset). Subsequently, a naïve logistic regression was performed along with the Akaike Information Criteria (AIC) to select the most parsimonious model (best model) in a stepwise backward algorithm by using the stepAIC function [25]. In the stepwise backward AIC, the selection process starts with a model including all variables of interest. At each step, a variable is excluded from the model if its elimination results in a higher AIC value than the previous model [25]. The best model is defined as the one with the lowest AIC value compared to the other possible explanatory models. Thirteen variables of interest were considered with their respective dummy variables resulting in 8,191 possible models. Additionally, a mixed-effect model was developed considering patients and health professionals as random intercepts and hence, accounting for the variance between- and within-patients and professionals on the outcome [26]. The glmer function was used to perform the mixed-effect model analyses [27]. To select the best mixed-effect model, a forward and backward stepwise algorithm was performed based on the AIC criteria. The intra-class correlation coefficient (ICC) [26] within-clusters was calculated for the best mixed-effect model to verify within-clusters dependency using the sjstats R package [28]. The best naïve model and the best mixed-effect model were compared, and the model with the smallest AIC value was considered the final best model. The variable importance in the best final model was estimated using the permute.varimp R function [29]. In this function, the values of each predictor are randomly permuted to break their association with the response, and the model is re-fit to a new dataset containing the permuted values [29]. The fit of the new model is compared to that of the original model [29]. The variables presenting the higher AICc difference between the original model and the model with the permuted predictor were considered as having higher importance in the model.

#### Akaike inflection criteria

The Akaike Information Criteria (AIC) was developed by Hirotugu Akaike to identify which combination of variables would best explain an outcome given a universe of potentially explanatory models [30]. The AIC is defined by the expression: AIC = - 2log (L (θ | y)) + 2K. Where, log (L (θ | y)) represents the maximum likelihood ratio (model quality), and K represents the number of variables included in the model (complexity) [30]. The best fit model is the one with the smallest possible number of parameters (parsimony) with a higher probability of explaining the outcome. This method is indicated to select models, which are developed based on observational data [30,31].

#### Model performance and validation

The area under the ROC curve (AUC) was used to assess the performance of the best model by using the pROC R package [32]. The AUC was calculated based on the training and the validation subsets and compared by using the roc.test function [32]. Additionally, the threshold that maximizes the sensitivity and specificity in classifying patients as no-show was identified.

#### Sensitivity analysis

A sensitivity analysis was conducted based on a model developed on 80% of the dataset (p80) and validated on the remaining 20% of the dataset to explore if it would improve the final best model performance. The model selection of the final best p80 model was carried out following the methodology applied to select the final best p50 model. The AUC’s of both best models were compared by using the roc.test function [32].

## Results

### Descriptive analysis of the dataset

Of the 57,586 scheduled appointments in the period, 70.7% (n = 40,740) fulfilled the inclusion criteria including 5,637 patients. The prevalence of no-show was 13.0% (n = 5,282). The mean age of the sample was 41 years (SD 23.2). Thirty percent of the appointments were scheduled by male (n = 12,219) and 82.1% (n = 33,442) by patients self-reported as white. Thirty-six percent of the sample was delivered as same-day appointments (n = 14,653). The mean age of the attendance group was 41.2 years (SD 23.3), and the mean age of the no-show group was 39.8 years (SD 22.0). The median of patient previous attendance was smaller in the no-show (4.0, IQR 7.0) group compared to the attendance group (5.0, IQR 8.0), whereas the median lead time was higher (14.0 days, IQR 17.0) in the no-show group compared to the attendance group (2.2 days, IQR 14.1)–Table 1.

**Table 1:** Descriptive analysis of the variables by the outcome.

	Total	Attendance	No-show
	N = 40,740	N = 35,458	N = 5,282
**Patients features**			
Age, mean (SD) years	41.0 (23.2)	41.2 (23.3)	39.8 (22.0)
Gender, male: n (%)	12,219 (30.0)	10,789 (30.4)	1,430 (27.1)
Race/ethnicity, White: n (%)	33,442 (82.1)	29,210 (82.4)	4,232 (80.1)
Patient previous attendance: median (IQR)	5.0 (8.0)	5.0 (8.0)	4.0 (7.0)
Patient previous same-day appointment: median (IQR)	2.0 (3.0)	2.0 (3.0)	1.0 (3.0)
**Time metrics**	** **	** **	** **
Lead time, days: median (IQR)	4.0 (15.0)	2.0 (14.0)	14.0 (17.0)
Waiting time, min: median (IQR)	28.0 (59.0)	27.0 (57.0)	34.0 (76.0)
Same-day appointment calculated: n (%)	14,653 (36.0)	14,335 (40.4)	318 (6.0)
**Health professionals**	** **	** **	** **
Nursing: n (%)	5558 (13.6)	4,582 (12.9)	976 (18.5)
General practitioner: n (%)	23,578 (57.9)	21,544 (60.8)	2,034 (38.5)
Dentist: n (%)	7,674 (18.8)	6,473 (18.3)	1,201(22.7)
Pharmacist: n (%)	44 (0.1)	28 (0.1)	16 (0.3)
Nutritionist: n (%)	495 (1.2)	367 (1.0)	128 (2.4)
Psychologist: n (%)	1,935 (4.7)	1365 (3.8)	570 (10.8)
Social worker: n (%)	763 (1.9)	570 (1.6)	193 (3.7)
Oral health technician: n (%)	693 (1.7)	529 (1.5)	164 (3.1)
**Types of appointment**			
User embracement: n (%)	1094 (2.7)	946 (2.7)	148 (2.8)
Same-day appointment: n (%)	9,021 (22.1)	8,886 (25.1)	135 (2.6)
Extra-same-day appointment: n (%)	7,252 (17.8)	7,121 (20.1)	131 (2.5)
Extra-scheduled appointment: n (%)	2,110 (5.2)	1,743 (4.9)	367 (6.9)
Dental urgency/emergency: n (%)	889 (2.2)	868 (2.4)	21 (0.4)
Extra-scheduled dental appointment: n (%)	1,289 (3.2)	1,102 (3.1)	187 (3.5)
Rapid HIV test: n (%)	14 (0.03)	10 (0.03)	4 (0.1)
First dental appointment: n (%)	546 (1.3)	468 (1.3)	78 (1.5)
Hypertension/Diabetes dental appointment: n (%)	7 (0.02)	5 (0.01)	2 (0.04)
Dental appointment: n (%)	4736 (11.6)	3,836 (10.8)	900 (17.0)
Pharmacist appointment: n (%)	43 (0.1)	28 (0.1)	15 (0.3)
Nutritionist appointment: n (%)	4 (0.01)	3 (0.01)	1 (0.02)
Psychologist appointment: n (%)	1,915 (4.7)	1,353 (3.8)	562 (10.6)
Social worker appointment: n (%)	721 (1.8)	531 (1.5)	190 (3.6)
Oral health technician appointment: n (%)	609 (1.5)	463 (1.3)	146 (2.8)
Prenatal health care program: n (%)	914 (2.2)	748 (2.1)	166 (3.1)
Child health care program: n (%)	1,292 (3.2)	1,002 (2.8)	290 (5.5)
Pap smear screening program: n (%)	2,229 (5.5)	1,472 (4.2)	757 (14.3)
Hypertension/Diabetes health care program: n (%)	1,841 (4.5)	1,464 (4.1)	377 (7.1)
Tuberculosis control program: n (%)	60 (0.1)	48 (0.1)	12 (0.2)
Adult health care program: n (%)	316 (0.8)	249 (0.7)	67 (1.3)
Return: n (%)	742 (1.8)	599 (1.7)	143 (2.7)
Individual appointment: n (%)	22 (0.1)	16 (0.05)	6 (0.1)
Elderly group: n (%)	1,755 (4.3)	1,557 (4.4)	198 (3.7)
Asthma control group: n (%)	474 (1.2)	342 (1.0)	132 (2.5)
Tabaco control group: n (%)	8 (0.02)	6 (0.02)	2 (0.04)
Mental health group: n (%)	406 (1.0)	310 (0.9)	96 (1.8)
Quality of life group: n (%)	431 (1.1)	282 (0.8)	149 (2.8)
**Appointment shift,** Morning: n (%)	23,091 (56.7)	20,186 (56.9)	2,905 (55.0)
**Appointment Weekday**			
Monday: n (%)	10,140 (24.9)	8,811 (24.8)	1,329 (25.2)
Tuesday: n (%)	8,236 (20.2)	7,147 (20.2)	1,089 (20.6)
Wednesday: n (%)	9,072 (22.3)	7,827 (22.1)	1,245 (23.6)
Thursday: n (%)	6,932 (17.0)	6,094 (17.2)	838 (15.9)
Friday: n (%)	6,291 (15.4)	5,526 (15.6)	765 (14.5)
Saturday: n (%)	69 (0.2)	53 (0.1)	16 (0.3)
**Appointment month**			
January: n (%)	4,128 (10.1)	3,651 (10.3)	477 (9.0)
February: n (%)	2,260 (5.5)	1,935 (5.5)	325 (6.2)
March: n (%)	2,780 (6.8)	2,460 (6.9)	320 (6.1)
April: n (%)	3,100 (7.6)	2,752 (7.8)	348 (6.6)
May: n (%)	3,384 (8.3)	2,976 (8.4)	408 (7.7)
June: n (%)	3,687 (9.1)	3,222 (9.1)	465 (8.8)
July: n (%)	3,304 (8.1)	2,852 (8.0)	452 (8.6)
August: n (%)	3,224 (7.9)	2,792 (7.9)	432 (8.2)
September: n (%)	3,108 (7.6)	2,717 (7.7)	391 (7.4)
October: n (%)	4,038 (9.9)	3,557 (10.0)	481 (9.1)
November: n (%)	3,493 (8.6)	2,982 (8.4)	511 (9.7)
December: n (%)	4,234 (10.4)	3,562 (10.0)	672 (12.7)

### Model development and selection

The backward stepwise algorithm compared all possible models based on 20,368 scheduled appointments (50% of the dataset). Two observations were excluded due to missingness in the waiting time variable. The stepwise procedure fitted forty models. The best naïve model presented an AIC value of 12,974. The best mixed-effect model presented an AIC value of 12,763 which was smaller than the naïve model and hence it was considered the best final model (p50). Table 2 presented the final best model results including the combination of variables that would best estimate the probability of no show, given a universe of potentially explanatory models. The most important variables in the p50 model were the type of appointment (difference in the AICc = 804), previous attendance (AICc difference = 281), previous same-day appointment (AICc difference = 114) and same-day appointment (AICc difference = 110). The variance within patients was 0.185 (SD 0.430) and within health professionals was 0.020 (SD 0.143). The intra-class correlation coefficient between patients was 0.05 and between health professionals was 0.003.

**Table 2:** Results of mixed-effect logistic regression of the final best model–p50.

	Regression coefficients	95% CI
Intercept	-1.193	(-1.193; -0.759)
**Patient features**		
Age	-0.007	(-0.010; -0.004)
Male	-0.018	(-0.132; 0.095)
White	-0.119	(-0.241; 0.004)
**Time metrics**		
Patient previous attendance	-0.097	(-0.109; -0.085)
Patient previous same-day appointment	0.163	(0.135; 0.190)
Lead time	0.004	(0.001; 0.007)
Waiting time	0.001	(0.001; 0.002)
Same-day appointment calculated	-1.091	(-1.306; -0.877)
**Types of appointment**		
User embracement	REF	
Same-day appointment	-1.951	(-2.334; -1.567)
Extra-same-day appointment	-2.200	(-2.568; -1.832)
Extra-scheduled appointment	0.160	(-0.176; 0.496)
Dental urgency/emergency	-2.041	(-2.931; -1.150)
Extra-scheduled dental appointment	-0.134	(-0.588; 0.320)
Rapid HIV test	0.048	(-2.244; 2.340)
First dental appointment	-0.194	(-0.716; 0.327)
HT/DM dental appointment	-10.720	(-41.162; 19.716)
Dental appointment	0.275	(-0.134; 0.683)
Pharmacist appointment	0.842	(-0.197; 1.880)
Nutritionist appointment	0.250	(-2.316; 2.815)
Psychologist appointment	0.867	(0.372; 1.361)
Social worker appointment	0.602	(0.110; 1.094)
Oral health technician appointment	0.589	(0.066; 1.112)
Prenatal health care program	0.102	(-0.270; 0.474)
Child health care program	0.308	(-0.028; 0.644)
Pap smear screening program	0.879	(0.573; 1.184)
HT/DM health care program	0.523	(0.208; 0.839)
Tuberculosis control program	0.097	(-1.051; 1.246)
Adult health care program	0.400	(-0.065; 0.866)
Return	0.303	(-0.072; 0.677)
Individual appointment	1.156	(0.011; 2.302)
Elderly group	0.096	(-0.246; 0.438)
Asthma control group	0.633	(0.226; 1.039)
Tabaco control group	1.499	(-1.408; 4.407)
Mental health group	0.660	(0.234; 1.085)
Quality of life group	0.889	(0.459; 1.319)
**Appointment shift**		
Afternoon	0.038	(-0.061; 0.137)
**Appointment weekday**		
Monday	0.145	(0.012; 0.277)
Tuesday	0.014	(-0.122; 0.151)
Wednesday	REF	
Thursday	-0.113	(-0.261; 0.035)
Friday	0.006	(-0.149; 0.161)
Saturday	0.250	(-0.524; 1.025)
**Appointment month**		
January	0.104	(-0.124; 0.333)
February	0.375	(0.121; 0.629)
March	0.135	(-0.116; 0.385)
April		
May	0.168	(-0.063; 0.398)
June	0.208	(-0.023; 0.439)
July	0.112	(-0.120; 0.344)
August	0.094	(-0.143; 0.331)
September	-0.099	(-0.339; 0.141)
October	-0.111	(-0.341; 0.118)
November	0.038	(-0.192; 0.269)
December	0.334	(0.118; 0.550)
**Day of the month**	**0.001**	**(-0.005; 0.006)**

Random effects: Patient variance = 0.1849, sd = 0.430; Professional variance = 0.020, sd = 0.143. sd: standard deviation. 95% CI: 95% confidence interval. REF: reference category.

### Model performance and validation

**Fig 1:**
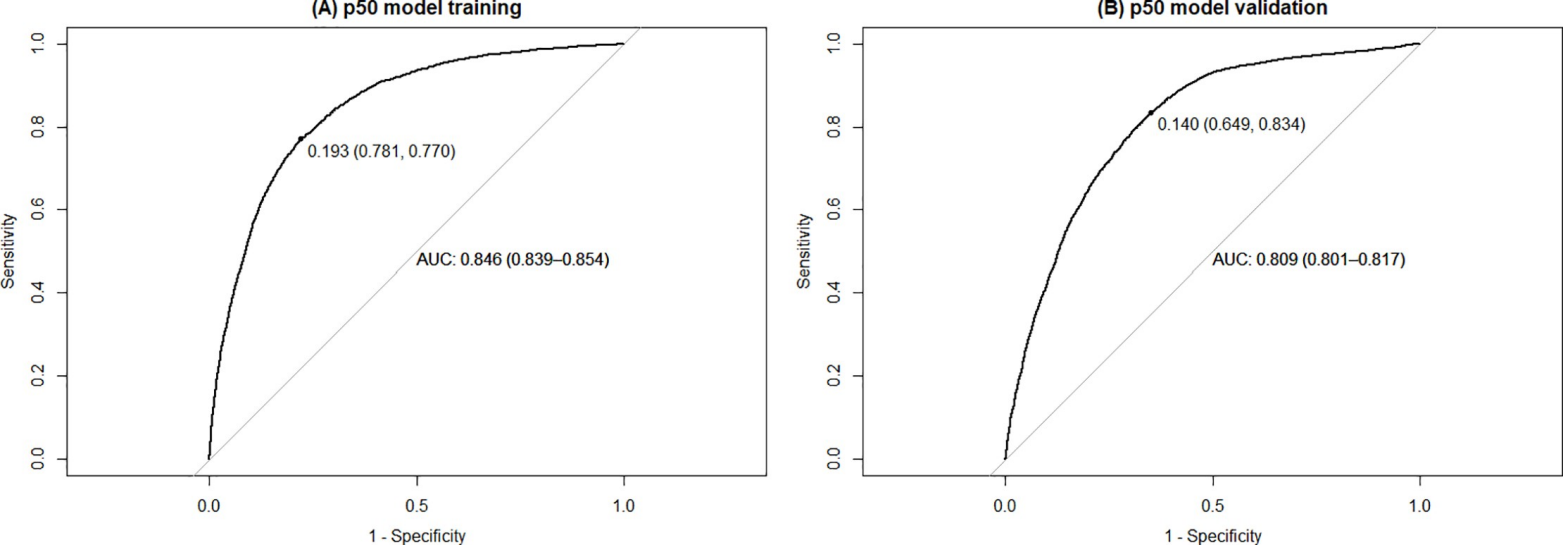
Performance of the patient no-show predictive model developed on 50% (p50) of the dataset. (A) shows the AUC (95% confidence interval) of the p50 model tested on the same subset from which it was developed (training subset). (B) shows the AUC (95% confidence interval) of the p50 model tested on the remaining 50% of the dataset (validation subset). The point in the curve is the threshold that maximizes the sensitivity and specificity of the model. The sensitivity and specificity are in the parenthesis. AIC: Akaike Information Criteria. AUC: area under the Receiver Operating Characteristic curve.

Table 3 presents the comparison between development and validation data. The AUC of the p50 model in the training subset was 84.6% (95% CI 83.9–85.4). The threshold of 0.193 presented the maximum sensitivity of 78.1% and specificity of 77.0% (Fig 1). When the model was validated on empirical data, the AUC was slightly lower 80.9% (95% CI 80.1–81.7) compared to the AUC of the training subset (Fig 1). This difference was statistically significant (*p*<0.001). The threshold of 0.140 presented the maximum sensitivity of 64.9% and specificity of 83.4% when the p50 model was validated. The high specificity of the model is preferable over high sensitivity, as it avoids staff work overload with false positives of no-shows in the case of overbooking.

**Table 3:** Comparison between development and validation data.

	Training data n = 20,370	Validation data n = 20,370
**No-Show: n (%)**	2,695 (13.2)	2,587 (12.7)
**Patients features**		
Age, years: mean (SD)	40.9 (23.2)	41.2 (23.1)
Sex, male: n (%)	6,149 (30.2)	6,070 (29.8)
Race/ethnicity, White: n (%)	16,787 (82.4)	16,655 (81.8)
Patient previous attendance: median (IQR)	5.0 (8.0)	5.0 (8.0)
Patient previous same-day appointment: median (IQR)	2.0 (3.0)	2.0 (3.0)
**Time metrics**		
Lead time, days: median (IQR)	4.1 (15.9)	4.0 (15.8)
Waiting time, min: median (IQR)	27.0 (58.0)	28.0 (59.0)
Same-day appointment calculated: n (%)	7,276 (35.7)	7,377 (36.2)
**Health professionals**		
Nursing: n (%)	2,818 (13.8)	2,740 (13.5)
General practitioner: n (%)	11,792 (57.9)	11,786 (57.9)
Dentist: n (%)	3,798 (18.6)	3,876 (19.0)
Pharmacist: n (%)	22 (0.1)	22 (0.1)
Nutritionist: n (%)	260 (1.3)	235 (1.2)
Psychologist: n (%)	962 (4.7)	973 (4.8)
Social worker: n (%)	369 (1.8)	394 (1.9)
Oral health technician: n (%)	249 (1.7)	344 (1.7)
**Types of appointment**		
User embracement: n (%)	557 (2.7)	537 (2.6)
Same-day appointment: n (%)	4,540 (22.3)	4,481 (22.0)
Extra-same-day appointment: n (%)	3,593 (17.6)	3,659 (18.0)
Extra-scheduled appointment: n (%)	1,075 (5.3)	1,035 (5.1)
Dental urgency/emergency: n (%)	429 (2.1)	460 (2.3)
Extra-scheduled dental appointment: n (%)	655 (3.2)	634 (3.1)
Rapid HIV test: n (%)	7 (0.003)	7 (0.003)
First dental appointment: n (%)	270 (1.3)	276 (1.4)
Hypertension/Diabetes dental appointment: n (%)	4 (0.02)	3 (0.01)
Dental appointment: n (%)	2,335 (11.5)	2,401 (11.8)
Pharmacist appointment: n (%)	21 (0.1)	22 (0.1)
Nutritionist appointment: n (%)	3 (0.01)	1 (0.005)
Psychologist appointment: n (%)	949 (4.7)	966 (4.7)
Social worker appointment: n (%)	351 (1.7)	370 (1.8)
Oral health technician appointment: n (%)	297 (1.5)	312 (1.5)
Prenatal health care program: n (%)	477 (2.3)	437 (2.1)
Child health care program: n (%)	659 (3.2)	633 (3.1)
Pap smear screening program: n (%)	1,135 (5.6)	1,094 (5.4)
Hypertension/Diabetes health care program: n (%)	900 (4.4)	941 (4.6)
Tuberculosis control program: n (%)	23 (0.1)	37 (0.2)
Adult health care program: n (%)	152 (0.7)	164 (0.8)
Return: n (%)	367 (1.8)	375 (1.8)
Individual appointment: n (%)	15 (0.1)	7 (0.03)
Elderly group: n (%)	890 (4.4)	865 (4.2)
Asthma control group: n (%)	250 (1.2)	224 (1.1)
Tabaco control group: n (%)	4 (0.02)	4 (0.02)
Mental health group: n (%)	188 (0.9)	218 (1.1)
Quality of life group: n (%)	224 (1.1)	207 (1.0)
**Appointment shift,** Morning: n (%)	11,500 (56.5)	11,591 (56.9)
**Appointment Weekday**		
Monday: n (%)	5,095 (25.0)	5,045 (24.8)
Tuesday: n (%)	4,223 (20.7)	4,013 (19.7)
Wednesday: n (%)	4,476 (22.0)	4,596 (22.6)
Thursday: n (%)	3,356 (16.5)	3,576 (17.6)
Friday: n (%)	3,185 (15.6)	3,106 (15.2)
Saturday: n (%)	35 (0.2)	34 (0.2)
**Appointment month**		
January: n (%)	2,028 (10.0)	2,100 (10.3)
February: n (%)	1,131 (5.6)	1,129 (5.5)
March: n (%)	1,377 (6.8)	1,403 (6.9)
April: n (%)	1,550 (7.6)	1,550 (7.6)
May: n (%)	1,668 (8.2)	1,716 (8.4)
June: n (%)	1,675 (8.2)	1,629 (8.0)
July: n (%)	1,896 (9.3)	1,791 (8.8)
August: n (%)	1,622 (8.0)	1,602 (7.9)
September: n (%)	1,562 (7.7)	1,546 (7.6)
October: n (%)	2,006 (9.8)	2,032 (10.0)
November: n (%)	1,769 (8.7)	1,724 (8.5)
December: n (%)	2,086 (10.2)	2,148 (10.5)

### Sensitivity analysis

The model p80 included all variables of the p50 model except the variable day of the month. When the p80 model was validated on empirical data, the AUC was 81.9 (95% CI 80.6–83.2). Despite the better performance, it was not statistically different compared with the AUC of the p50 model 80.9% (95% CI 80.1–81.7).

### Practical application of the predictive patient no-show model

A patient of 20 years-old, non-white, 0 previous attendance and 1 previous same-day appointment scheduled an appointment with the psychologist within 14 days (appointment weekday and month: Monday and March, respectively). The probability of patient-no-show, according to the p50 model was 0.591. Based on the threshold of 0.140, the patient would be classified as a no-show. If an appointment is overbooked in this slot, the model would have a probability of 81% to correctly identify the true positives and negatives of no-show–S1 File.

## Discussion

This study explored the factors associated with no-show at a primary care setting in Southern Brazil and developed and validated a patient no-show predictive model based on empirical data. It revealed that previous patient attendance and same-day appointments were the most important predictors of a no-show in the service investigated. More importantly, the results showed that the best model, developed from data already available in the scheduling system, had a good performance with a probability of 81% to correctly identify the true positives and negatives of a patient no-show.

Alike previously published models, our results revealed previous patient attendance as one of the most important predictors of no-show [18–21,33]. Aware of this, Harris et al. developed a predictive no-show model including solely the patient’s past attendance history, observing an accuracy around 0.70 [21], which is slightly lower than our best model and other models with additional factors (i.e., sociodemographic and medical background). Nevertheless, it is difficult to compare the performance of all these models because they (1) were based on different population, (2) included different predictors (i.e., marital status, religion, socioeconomic status, insurance coverage, and comorbidities), (3) used different modelling techniques and (3) considered different methods to estimate patient past attendance history. However, their performances were very similar to what we found, ranging from 0.69 to 0.82 [18–21,33].

Our study differs from the previous models because we used a mixed-effect modelling approach to account for the variance across patients and health professional and developed relatively simple models and compared them using a multimodel inference method. The AIC allows selecting models considering the strength of evidence and uncertainty in the selection process [34]. This information-theoretic approach has been deemed more appropriate to deal with the complexity of the real world problems and has been mainly used by biologists [34]. In this method, the goal is to identify the best fit model given the data available, which is quite different than finding full truth [34,35]. Given the complexity of the no-show issue, which encompasses several factors, the final best model probably did not include all universe of variables to explain the outcome. Aware of this, our study had conducted further testing and, hence, observing a good performance of the final best model to predict patient no-show when validated on empirical data.

Based on the results of this study, one could explore the potential of incorporating the patient no-show predictive model into the scheduling system of the service, which might aid overbooking approaches in programs associated with high non-attendance rates (i.e., Pap smear screening or Hypertension/Diabetes health care program). Additionally, adopting overbooking based on patient no-show predictive models, instead of using only a flat non-attendance prevalence, would avoid false positives and hence avoid excessive work load for the healthcare team [17]. As expected, we found that same-day appointment is less likely of non-show than scheduled appointment ahead of time. In a systematic review, Ansell et al. [36] found that no-show rates decreased after the implementation of same-day appointments, further referred to as “open access scheduling.” The rationale of the open access approach is that patients would have access to care in the time when they need most. On the other hand, open access scheduling may overload the health care staff if the demand exceeds the supply, which could compromise the quality of care [37]. Hitherto, its implementations would require at least a resizing of the service’s patient load [37] and an evaluation of its impact on quality of care [36], which are beyond the scope of this study. However, our results may provide some insight about the daily agenda optimization, aiming at reaching a balance between same day-appointments and advanced-scheduled ones.

This study has some limitations. Despite the advantage of the stepwise algorithm of comparing predictors automatically, it may lead to spurious associations [25,38]. Taking this into account, we focused on select no-show predictors reported on literature and on the experience of the primary health care team instead of just relying on the algorithm’s choice. Another issue regards to missing data excluded from the analysis. However, we assume the missingness was related to data registration issues and not associated with variables of interest. Hence, the exclusion of missing cases is known to produce unbiased estimates and conservative results [39]. Also, we did not include sociodemographic statuses such as educational level and income due to missingness in the database. Further investigation should explore if these inputs improve the performance of predictive no-show models.

## Conclusions

This study developed and validated a patient no-show predictive model based on data from a public primary care setting in Southern Brazil. It mainly revealed that using the information already available in the scheduling system, the best fit model presented a good performance to predict no-show when empirically validated. Additionally, the methodology applied in this study may be useful to other health care services to develop predictive no-show models based on their specific population. It is expected this approach to be helpful to overbooking decision in scheduling systems. Further investigation is needed to explore the effectiveness of using this model in terms of improving service performance and its impact on quality of care compared to the usual practice.

## Supporting information

S1 Table Types of appointment: Definition based on the primary care service database. (DOCX)

S1 File No-show predictive model calculator. Practical application of the predictive patient no-show model.
(XLSX)
