# Supplementary material for: Development and validation of a patient no-show predictive model at a primary care setting in Southern Brazil
Source: PLoS One. 2019 Apr 4;14(4):e0214869. doi: 10.1371/journal.pone.0214869 (PMC6448862; doi:10.1371/journal.pone.0214869)
Supplement: S1 Table — (DOCX) [file pone.0214869.s001.docx]

**S1 Table. Types of appointment: definition based on the primary care service database**

| **Type of appointment** | **Definition** |
| --- | --- |
| **User embracement** | It is a same-day appointment scheduled in the professionals’ agenda to attend daily demand of the service |
| **Same-day appointment** | Any appointment scheduled and held on the same day |
| **Extra-same-day appointment** | It is a same-day appointment worked into the health care professionals’ agenda to attend patients who come to the service without a previously scheduled appointment. In this case, there is no more urgent/emergency appointment available in the professionals’ agenda, but the patient obtains an appointment anyway |
| **Extra-schedule appointment** | It is an appointment worked into the professionals’ agenda to attend patients who come to the service without a previously scheduled appointment. In this case, there is no more user embracement appointment available, but the patient is attended to on another day |
| **Dental urgency/emergency** | It is a same-day appointment scheduled on the dentist's agenda to attend people with urgent/emergency dental problems |
| **Extra-scheduled dental appointment** | It is a same-day appointment worked into the dentist agenda to attend patients who come to the service without a previously scheduled appointment. In this case, there is no more dental urgency/emergency appointment available, but the patient is attended to anyway |
| **Rapid HIV test** | It is a same-day appointment scheduled in the nursing agenda to perform Rapid HIV test |
| **First-dental appointment** | First appointment with the dentist |
| **HT/DM dental appointment** | Appointment schedule on the dentist's agenda for people with Hypertension or Diabetes |
| **Dental Appointment** | Appointment scheduled in the dentist’s agenda |
| **Pharmacist appointment** | Appointment scheduled in the pharmacist’s agenda |
| **Nutritionist appointment** | Appointment scheduled in the nutritionist’s agenda |
| **Psychologist appointment** | Appointment scheduled in the psychologist’s agenda |
| **Social worker appointment** | Appointment scheduled in the social worker’s agenda |
| **Oral health technician appointment** | Appointment scheduled in the oral health technician’s agenda and offered to provide guidance related to oral health |
| **Prenatal health care program** | Appointment scheduled in the nursing or the general practitioner agenda and offered to pregnant women |
| **Child health care program** | Appointment scheduled in the nursing or the general practitioner agenda for children up to 12 years |
| **Pap smear screening program** | Appointment scheduled in the nursing or the general practitioner agenda and offered to women from 25 to 64 years to screen for cervical cancer |
| **HT/DM health care program** | Appointment scheduled in the nursing or the general practitioner agenda and offered to people with hypertension and diabetes |
| **Tuberculosis control program** | Appointment scheduled in the nursing or the general practitioner agenda and offered to people with tuberculosis |
| **Adult health care program** | Appointment scheduled in the nursing or the general practitioner agenda and offered to adults |
| **Return** | Appointment scheduled in the professional’s agenda for the reassessment of a patient |
| **Individual appointment** | Individual appointment carried out by any health care professional |
| **Elderly group** | Group activity scheduled in the health care professional’s agenda and offered to the elderly population to discuss health issues |
| **Asthma control group** | Group activity scheduled in the health care professional’s agenda and offered to people with asthma to discuss health issues |
| **Tobacco control group** | Group activity scheduled in the health care professional’s agenda and offered to people with a dependence on tobacco |
| **Mental health group** | Group activity scheduled in the health care professional’s agenda and offered to people with mental health problems |
| **Quality of life group** | Group activity scheduled in the health care professionals’ agenda and offered to the general population |
